# Results and Methodological Implications of the Digital Epidemiology of Prescription Drug References Among Twitter Users: Latent Dirichlet Allocation (LDA) Analyses

**DOI:** 10.2196/48405

**Published:** 2023-07-28

**Authors:** Maria A Parker, Danny Valdez, Varun K Rao, Katherine S Eddens, Jon Agley

**Affiliations:** 1 Department of Epidemiology and Biostatistics School of Public Health Indiana University Bloomington Bloomington, IN United States; 2 Department of Applied Health Science School of Public Health Indiana University Bloomington Bloomington, IN United States; 3 Department of Informatics Luddy School of Informatics, Computing, and Engineering Indiana University Bloomington Bloomington, IN United States

**Keywords:** Twitter, LDA, drug use, digital epidemiology, unsupervised analysis, tweet, tweets, social media, epidemiology, epidemiological, machine learning, text mining, data mining, pharmacy, pharmaceutic, pharmaceutical, pharmaceuticals, drug, prescription, NLP, natural language processing

## Abstract

**Background:**

Social media is an important information source for a growing subset of the population and can likely be leveraged to provide insight into the evolving drug overdose epidemic. Twitter can provide valuable insight into trends, colloquial information available to potential users, and how networks and interactivity might influence what people are exposed to and how they engage in communication around drug use.

**Objective:**

This exploratory study was designed to investigate the ways in which unsupervised machine learning analyses using natural language processing could identify coherent themes for tweets containing substance names.

**Methods:**

This study involved harnessing data from Twitter, including large-scale collection of brand name (N=262,607) and street name (N=204,068) prescription drug–related tweets and use of unsupervised machine learning analyses (ie, natural language processing) of collected data with data visualization to identify pertinent tweet themes. Latent Dirichlet allocation (LDA) with coherence score calculations was performed to compare brand (eg, OxyContin) and street (eg, oxys) name tweets.

**Results:**

We found people discussed drug use differently depending on whether a brand name or street name was used. Brand name categories often contained political talking points (eg, border, crime, and political handling of ongoing drug mitigation strategies). In contrast, categories containing street names occasionally referenced drug misuse, though multiple social uses for a term (eg, Sonata) muddled topic clarity.

**Conclusions:**

Content in the brand name corpus reflected discussion about the drug itself and less often reflected personal use. However, content in the street name corpus was notably more diverse and resisted simple LDA categorization. We speculate this may reflect effective use of slang terminology to clandestinely discuss drug-related activity. If so, straightforward analyses of digital drug-related communication may be more difficult than previously assumed. This work has the potential to be used for surveillance and detection of harmful drug use information. It also might be used for appropriate education and dissemination of information to persons engaged in drug use content on Twitter.

## Introduction

### Background

The drug overdose epidemic has claimed more than 100,000 lives in 12-month year/year mortality reports for the past several years [1]. The epidemic is constantly changing and has arguably done so at least 4 significant times (“waves”) since 2002 [2]. The current (fourth) wave involves high mortality from stimulants and illicit fentanyl, both through unintentional ingestion of fentanyl (eg, as a contaminant) and from comorbid use with other drugs [2]. However, in drug use research and in public perception, individual drugs and drug use disorders are often investigated in isolation (eg, a perception that the current epidemic remains an “opioid use” crisis), even though many individuals use drugs in combination or may not even intend to ingest an opioid prior to opioid overdose. These concerns are borne out in overdose death records. For example, an alarming increase in deaths involving methamphetamine and cocaine, with overdoses of both exceeding 10,000 in year/year estimates, illustrates that the current crisis may be more appropriately characterized as a polydrug overdose crisis [3].

### Literature Review

Social media is an important information source and communication tool for a growing subset of the population. The Pew Research Center [4] estimates that 84% of people ages 18 years to 24 years use at least one social media site. Increasingly, the idea that we gather in a “virtual town square” [5] is borne out in reality. It is therefore unsurprising that drug use—in a wide variety of forms and manners—is discussed on platforms like Twitter openly and without perceived judgement. In many ways, this reflects our nation’s long history and current interest in all things psychoactive [6]. It also logically follows that social media is an emerging venue for observational research on drug use. For example, individuals participate in online communities, social relationships, and conversations about drug use (including transactional dialogue) on Twitter, which may be leveraged to provide insight into this evolving epidemic [7-9]. Twitter (and presumably platforms operating in a similar manner) can provide valuable insight into trends in discourse, the types of colloquial information available to potential drug users, and how networks and interactivity might influence content exposure and expression. Research has described Twitter’s potential for serving as a platform for real-time content analysis [10], and it has been extensively used to study a multitude of mental health phenomena, including resilience, internalizing disorders, and help-seeking behaviors [11-13]. Twitter can provide valuable insight into trends (eg, via predictive analytics), colloquial information available to potential users (eg, via natural language processing [NLP]), and how networks and interactivity might influence the content to which people are exposed and that they express (eg, via social network analysis) [7].

Although there have been numerous studies that explore one drug or drug class, such as prescription drug misuse, in the Twittersphere [7,14,15], there have been fewer related to polydrug use [7,16,17] or drug use more broadly [18,19]. Additional research is needed to leverage “infoveillance” strategies for drug-related content on social media. Digital epidemiology can be used to help us identify themes online [20,21]. Discussion of drug use and overdose via Twitter is common and may offer insights into how drugs are shared or discussed. Indeed, querying Twitter data for specific keywords associated with a drug’s prescription or street name yields large-scale data sets with such potential insights. However, these data sets require appropriate processing, analyses, and visualization to assess potential behavioral risk factors and communication patterns and to facilitate interpretation.

In the specific area of overdose deaths, the foundational components needed to conduct rigorous social media research are not currently in place. The breadth and complexity of the ways in which drug use is discussed in formal and informal ways mean that contextually naïve approaches to social media analyses (eg, those that are not informed by topic-specific expertise in drug epidemiology) may struggle to produce meaningful and coherent output. This study lays out preliminary analyses and decision-making heuristics developed by a multidisciplinary team of researchers with expertise in big data analyses, social media, communication networks, and drug epidemiology. We leveraged Twitter’s application programming interface (API) to longitudinally scrape content specific to drug use, polydrug use, and overdose. An advantage of this approach is that Twitter’s API can provide access to a large number of discrete “documents” (ie, individual tweets) with limited character length, meaning that the number of possible “topics” appearing in any given tweet is limited by the nature of the platform.

For this study, we were interested in studying the digital ecosystem for drug-related communication in this social media space. We suspected that the manifestations of drug-related content might also differ meaningfully depending on whether “brand” or “prescription” drug terms were used to identify tweets or whether “street names” were used to identify tweets. Brand or prescription terms may be used to discuss social events or things that are observed, whereas street names logically may be intended to conceal the topic of discussion or to signal group membership (though to the degree that they enter popular discourse, these purposes may shift). As formative research, this study was guided by the following 3 research questions:

1. What themes emerged from a corpus of tweets containing references to brand or prescription-named drugs (eg, OxyContin and oxycodone)?
2. What themes emerged from a corpus of tweets containing references to street names of prescription medications (eg, oxys, oxi)?
3. What differences could be observed between themes identified from brand versus street names of the same prescription drugs?

Findings from this exploratory study stand to inform the relative landscape of digital communication relating to drug use and drug sharing online (ie, not tobacco or alcohol related). Insights from this study can be further leveraged to inform digital intervention and policy work and to provide further methodological considerations for conducting this type of digital epidemiology.

## Methods

### Ethical Considerations

This study was reviewed by the Indiana University Institutional Review Board (protocol #18081) and received an exempt designation.

### Data Collection

Data for this study were collected over a 3-month period from the public Twitter API using strategic queries and Boolean phrases (eg, OR, AND operators) to elicit data pertaining to drug-related communication on Twitter. These phrases were used to create a composite data set that was saved into a secure repository. Personally identifiable information was removed ahead of formal analysis. We identified appropriate queries by leveraging the National Institute on Drug Abuse website for commonly used drugs and their colloquial terms (or street names) [22]. Using this list, we developed keywords as filters that were then applied to the collection of Twitter data [15]. Keywords included a drug’s generic name (eg, oxycodone), brand name (eg, OxyContin, Xanax), and “street” names (eg, oxy, xannies).

To create a comparative study, we created 2 distinct corpora: (1) a corpus of tweets containing references to brand names of prescription drugs (hereafter referred to as the Brand Names Corpus) and (2) a corpus of tweets containing references to street names of prescription drugs (hereafter referred to as the Street Name Corpus). Our final sample sizes after data cleaning, screening for duplicates, and other irrelevant data yielded 262,607 tweets in the Brand Names Corpus (Nbrand_name) and 204,068 tweets in the Street Name Corpus (Nstreet_name) for a final sample size of 466,675 tweets.

### Analyses

#### LDA Topic Models

LDA is a commonly applied unsupervised NLP tool used to explore large-scale, unstructured corpora. LDA’s calculus for deriving a series of mathematically supported topics about a corpus has been colloquially described as a “bag of words” modeling approach [23] because it is unconcerned with sentence structure or word order and only focuses on the total set of words (“terms”) that exists within each tweet. To perform this analysis, there are 3 main entities of interest: words, documents, and a corpus. A document contains a specific sequence of words, and a set of documents is considered to be part of a corpus. Before any analysis is done, words that do not contribute to the document’s meaning must be removed. Since LDA identifies patterns via co-occurrences of different words, we are especially interested in words that have at least one independent semantic meaning. Thus, words that are punctuation marks, stop words, and hyperlinks were removed from each document. After those words were removed, we ran the LDA topic model to find the underlying topic model structure for each corpus.

**Figure 1:**
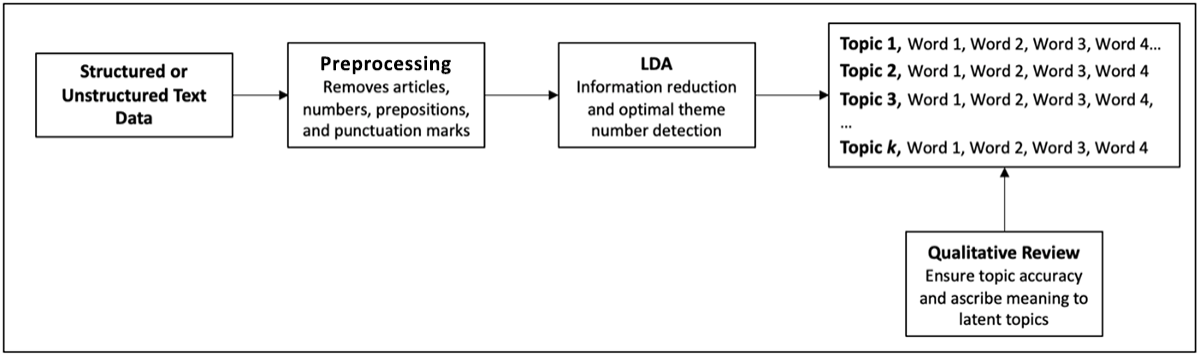
Conceptual diagram outlining a latent Dirichlet allocation (LDA) pipeline from preprocessing through qualitative review.

More formally, LDA is defined as a generative probabilistic model of a corpus [24]. In other words, documents are mixtures over a random distribution of topics, and each topic is represented via a distribution of words. Each document contains a set of words in which each word is distributed over a set of topics. According to Blei et al [24], a topic can be defined as a distribution over some fixed vocabulary. To approximate latent topics, LDA uses Bayesian modeling with Gibbs sampling, a calculation that compares every word “x” with every other word “y” across a series of “d” documents in a corpus. Words and terms with high degrees of co-occurrence, that is words and phrases that commonly appear together, are grouped to form a latent topic, which represents a core idea within the corpus. Although LDA identifies “topics” (sets of related terms) within the data set, the algorithm does not interpret what those topics mean. By viewing sets of terms used to form each topic and by reading example tweets that are strongly allocated to those topics, it is possible for researchers to label and assess the meaning of the topics generated by LDA. The utility of this approach is that it effectively leverages very large data sets: Although the allocation of any individual tweet may be either precise or imprecise, the overall generation of topics provides an accurate “10,000-mile“ overview of the co-occurrence of terms being used within the total corpus of documents. Figure 1 outlines a general LDA pipeline.

LDA topic models have been widely applied in the health and medical sciences in exploratory capacities to study the structure of related and unrelated documents. Although we acknowledge LDA’s age and function relative to newer, supervised NLP analyses (eg, BERT), we chose LDA due to its ease of access for non-NLP experts and its general application as an exploratory data analysis tool. For more information on topic modeling in health science, including proposed applications and functions, see studies by Valdez et al [13] and Valdez and Goodson [25].

#### Coherence Score Cross-Validation

Coherence score cross-validation refers to an iterative analysis to identify the optimal number of topics for a given corpus. Coherence score cross-validation was completed by programming a computer to iteratively run an LDA topic model for an increasing *k* number of topics. For each analysis, the computer generates a coherence score, which refers to the degree to which a topic can be accurately interpreted by a human. It is recommended that any exploratory analyses that utilize LDA topic models should report coherence scores as a measure of model fit. For more information on coherence scores and cross-validation, see [26].

#### Informal Qualitative Review

Although a computer can derive latent topics from a corpus, a computer cannot infer deeper meaning behind the content of the topics and what each topic represents. Thus, we performed an informal review, defined as an independent review and subsequent discussion, in which a randomly selected number of tweets per topic was reviewed by the research team to ascertain meaning. For more information on the qualitative review of NLP output, see Valdez et al [27].

### Procedure

**Figure 2:**
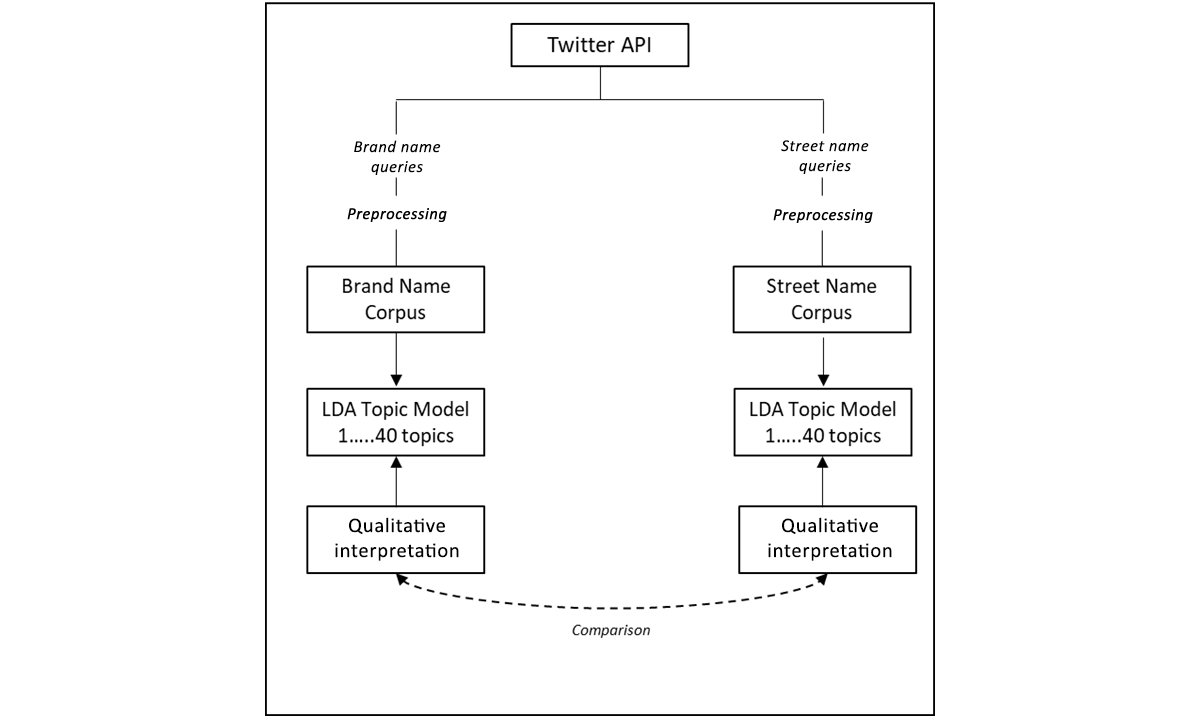
Conceptual diagram detailing our analysis pipeline. API: application programming interface; LDA: latent Dirichlet application.

Our workflow is depicted in Figure 2. First, we queried the Twitter API for top brand and street names commonly used by the US population. Tweets pertaining to brand name medications were triaged into the first corpus, and tweets pertaining to common street names were triaged into a second corpus. To pre-process the data, we ignored all articles and prepositions using the stopwords provided in the Natural Language Toolkit library. References to hyperlinks were ignored as well as white text and the @ symbol along with an individual's Twitter handle and all retweets. Duplicate tweets by the same user were also ignored, as well as any words that contained references to numbers. After pre-processing the data, the cleaned documents were used in the topic model. Any further references to tweets in this paper will refer to the uncleaned tweets, to provide the full context of the tweet. Then, we performed an iterative LDA on the corpora to determine the optimal number of topics. Once optimal topic numbers were identified, we ran a final LDA comprised of 20 topics for the Brand Name Corpus and 35 topics for the Street Name Corpus. Once we created topics, we used a “sort” function to classify all tweets in either corpus into one of the latent topics. The researchers on this team then convened to review a random selection of 25 to 50 tweets per topic to denote potential underlying meaning. Note, that because the “sort” function relies on keywords, rather than supervised sorting, there is typically a high degree of overlap among topics. As such, team members reviewed tweets per topic until unanimous agreement was reached regarding meaning [23].

## Results

### Overview

Our findings illustrate the digital ecosystem pertaining to prescription medication communication on Twitter. Across the Brand Name Corpus and Street Name Corpus, we observed several overarching themes that may offer insight into how drug use communication occurs online. However, there were also several noteworthy differences between corpora that may reinforce the difficulty of digital surveillance regarding drug use. We present our findings in the following sections, which are parsed by the outlined research questions.

### Aim 1: What Themes Emerged From a Corpus of Tweets Containing References to Brand or Prescription-Named Drugs?

**Figure 3:**
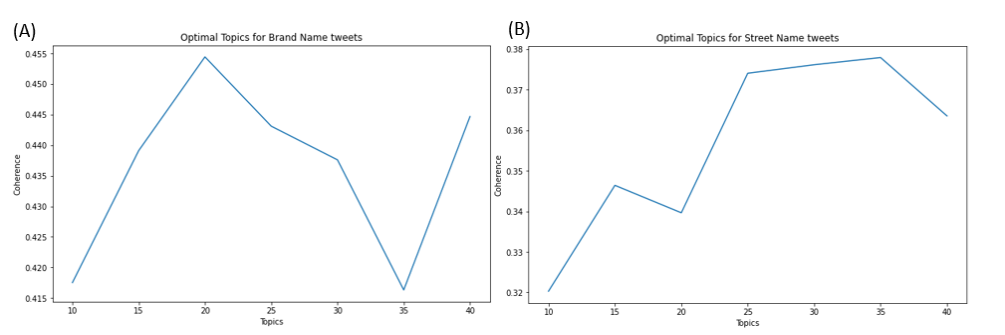
Coherence score plot for the iterative latent Dirichlet allocation (LDA) analyses across the (A) Brand Name Corpus and (B) Street Name Corpus.

For the Brand Name Corpus, our coherence score cross-validation indicated a 20-topic solution (Figure 3). Table 1 outlines each topic, with representative keywords and the total number of documents (or tweets) that was sorted per topic. From Table 1, we note that topics 1, 8, 10, 12, and 20 were the 5 topics with the largest documents per topic. Broadly, these topics represented the most frequently co-occurring themes embedded within the brand name data set and are the most dominant topics in 49.44% (129,832/262,607) of all documents in the data set. We observed that tweets related to the border, opioid crisis, and political figures were found in nearly every topic, which may suggest drug communication. Thus, as it relates to brand names, topics may principally frame drug use as an ongoing social issue. Although some topics did allude to general drug use in lighthearted, humorous, or other social contexts (eg, topics 19 and 10), these topics contained a minority of all the documents in the data set. From Table 2, we observe that the drugs that were discussed the most were Adderall, prescription drugs (Ritalin, Xanax, Valium), benzodiazepines, and fentanyl. Individuals discussing Adderall either discussed the general effects of using the drug (eg, tweet: I never really take my full Adderall dosage but lately I have for work and it has been so helpful lmao I truly do get in my own way), humorously discussed Adderall usage (eg, tweet: I can take multiple adderalls without problem but let me take a single adderall with a latte and I start getting scared lmao), or expressed concern over the Adderall shortage that began in October 2022 (eg, tweet: RT @BostonGlobe: Amid the Adderall shortage, people with ADHD face withdrawal and despair) [28]. The theme that had the largest number of topics was the theme with topics that referenced fentanyl use.

**Table 1:** Top 10 words in each topic for the Brand Name Corpus (N=262,607).

Topic ID	Keywords^a^	Documents per topic, n, %
1	adderall, get, shortage, eat, go, sh*t^b^, need, snort, let, trump	32,215 (12.27)
2	mdma, test, show, video, therapy, see, mind, meet, alcohol, think	7777 (2.96)
3	fentanyl, child, pill, report, find, fake, public, police, lace, candy	7163 (2.73)
4	new, thank, high, year, happy, record, beat, release, share, build	3895 (1.48)
5	sonata, watch, fit, car, piece, movement, winter, major, love, op	13,011 (4.95)
6	kid, sound, keep, really, truth, get, think, school, d*mn, parent’	6111 (2.32)
7	come, fight, go, eye, bar, future, real, couple, destroy, plan’	4052 (1.54)
8	fentanyl, border, kill, people, death, country, stop, cartel, die, crisis	32,536 (12.41)
9	get, ask, run, leave, say, guy, front, mom, find, go	7847 (2.99)
10	valium, take, m, go, xanax, need, know, get, talk, want	20,993 (7.99)
11	give, back, dose, turn, go, addiction, time, throw, face, catch	6705 (2.56)
12	adderall, feel, get, ritalin, adhd, work, take, make, prescribe, hard	24,148 (9.21)
13	adderall, pop, crazy, b*tch, feel, game, fly, finally, go, win	4716 (1.80)
14	make, weed, use, shroom, drug, fun, post, mdma, trip, available	7935 (3.02)
15	ambien, need, play, piano, listen, write, tweet, music, read, song	9557 (3.63)
16	good, never, man, ever, get, hear, bad, vicodin, life, think	11,981 (4.55)
17	drug, fentanyl, overdose, die, sell, cocaine, dealer, use, heroin, addict	16,462 (6.27)
18	adderall, help, use, people, also, anxiety, lot, lol, effect, drug	13,338 (5.09)
19	s, get, buy, prescription, doctor, oxycontin, week, month, pain, last	12,311 (4.67)
20	take, adderall, day, sleep, time, hour, today, night, drink, morning	19,846 (7.56)

^a^Sorted by word weight; weights correspond to word order.

^b^*Asterisks were added during the paper write-up but did not appear in the actual keywords.

**Table 2:** Brand name topics (n=20).

Topic theme	Topic IDs	Cumulative amount, n (%)
Adderall use or shortage	1, 5, 6, 12, 20	5 (25)
Adderall shortage	7, 13	2 (10)
Politics fentanyl	3, 8, 9, 15, 16, 17	6 (30)
Prescription drug use (Xanax, Valium, and Ritalin were all mentioned)	2, 4, 10	3 (15)
Stimulant use or lifestyle	11, 14, 19	3 (15)
Assorted drug use	18	1 (5)

**Figure 4:**
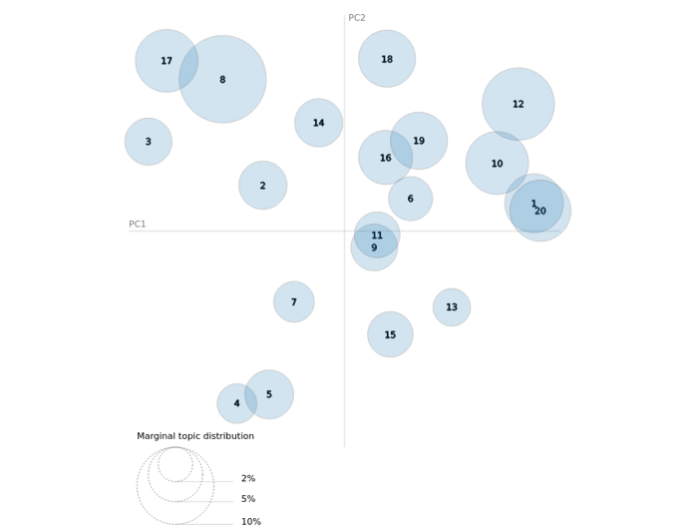
Intertopic distance map (via multidimensional scaling) for brand name tweets. PC: principal component.

Figure 4 presents an intertopic distance map, which broadly determines the relative similarities and differences across topics based on their word distributions [29]. A dynamic version of this figure is available online [30]. The figure implies that topics in the Brand Name Corpus were typically, but not always, distinct. From this figure, we can observe how interrelated and distinct certain topics are. For example, we find that topics 1 and 20 are very similar to one another, since they are plotted on top of one another, which is confirmed in Table 3, where we find topics 1 and 20 both reference Adderall use. Generally, topics that are close to one another have similar themes since these topics will have similar word distributions. However, even though topics can have similar word distributions, they can display different themes based on the weighting of certain words in those distributions. For example, topics 9 and 11 have similar words used in both topics but have different themes.

**Table 3:** Top 10 words in each topic for the Street Name Corpus (N=204,068).

ID	Keywords^a^	Documents per topic, n (%)
1	dog, cause, hear, get, light, mix, pain, go, benzo, problem	3783 (1.85)
2	man, good, morning, back, big, sorry, suck, well, here, look	3828 (1.88)
3	oxy, great, trade, stock, day, buy, today, ready, oil, trank	5571 (2.73)
4	play, team, top_analyst, price_target, week, vike, next_week, player, good, season	4756 (2.33)
5	start, fire, go, power, get, make, poor, skippy, money, office	3660 (1.79)
6	skippy, d*mn^b^, go, get, right, s, sit, brother, keep, tell	8876 (4.35)
7	downer, away, dexy, open, nice, upper, buy, room, release, go	3662 (1.79)
8	lol, name, smoke, week, sick, action, member, rule, crack, carry	2119 (1.04)
9	say, s, word, pull, guess, link, crystal, suppose, date, m	2939 (1.44)
10	love, much, go, really, get, m, sleep, fr, hope, help	5215 (2.56)
11	thank, hit, always, fan, wait, come, good, cute, still, funny	3077 (1.51)
12	cool, get, one, worth, hook, foot, fail, report, read, opinion	2240 (1.10)
13	take, watch, go, tonight, f*cking, welcome, benzo, get, usually, week	2871 (1.41)
14	drug, get, pill, take, smart, people, make, give, benzo, doctor	9527 (4.67)
15	know, hard, find, crystal_meth, vote, look, get, right, go, try	4256 (2.09)
16	downers_grove, do, cook, north, live, gt, south, company, day, el_rushbo	2375 (1.12)
17	use, new, year, dumb, happy, barb, seem, art, thing, also	3771 (1.85)
18	feel, happy, m, pill, make, take, eat, day, good, list	6121 (3.00)
19	leave, alone, get, stand, write, step, learn, rock, house, enough	2511 (1.23)
20	people, re, know, want, skippy, say, s, think, get, wrong	9732 (4.77)
21	percs, perc, get, pop, sh*t, f*ck, take, b*tch, go, fake	20,521 (1.01)
22	true, lose, hydro, performance, finish, good, attention, replace, mate, lucky	1604 (0.79)
23	barb, go, tweet, friend, follow, see, say, know, lie, happen	10,415 (5.10)
24	vike, game, go, win, get, bill, skol, viking, let, lay	19,027 (9.32)
25	bring, enjoy, lude, story, work, get, food, full, send, provide	3387 (1.66)
26	next, miss, move, check, day, make, video, group, hour, gain	2151 (1.05)
27	year, get, upper, last, old, long, home, fixer_upper, later, go	5425 (2.66)
28	need, happy_pill, stop, book, take, get, smile, together, seriously, always	4959 (2.43)
29	time, oxy, free, ahead, spend, levels_poste, family, market, never, break	2177 (1.07)
30	sure, drug, country, future, fentanyl, war, speed, police, business, arrest	2917 (1.42)
31	stay, vitamin, wish, school, ass, forget, beautiful, sexy_dexy, special, healthy	2640 (1.29)
32	way, benzo, get, help, omg, s, sound, test, sell, addicted	4032 (1.98)
33	yellow, red, blue, see, give, peanut_butter, card, green, goal, match	4367 (2.14)
34	barb, get, nicki, say, talk, think, literally, hate, even, g	10,790 (5.29)
35	upper, month, low, clean, barb, gun, get, high, lower, fit	2905 (1.42)

^a^Sorted by word weight; weights correspond to word order.

^b^*Asterisks were added during the paper write-up but did not appear in the actual keywords.

### Aim 2: What Themes Emerged From a Corpus of Tweets Containing References to Street Names for Prescription Medications?

**Figure 5:**
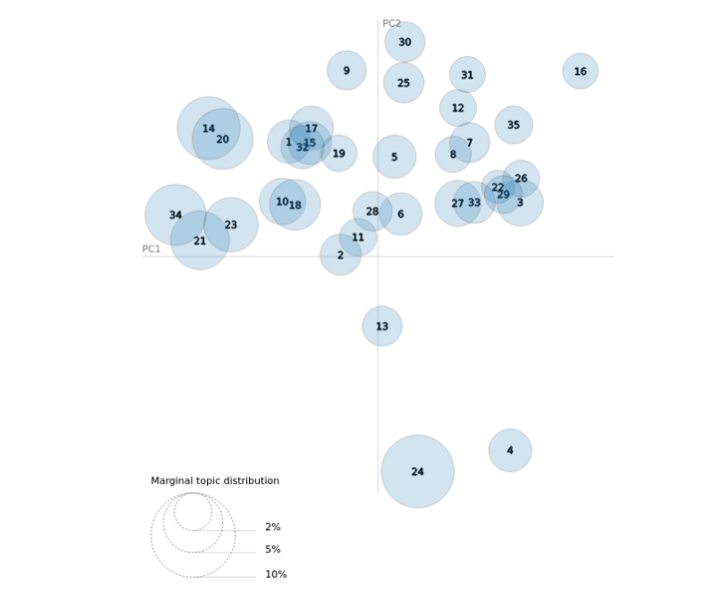
Intertopic distance map (via multidimensional scaling) for street name tweets. PC: principal component.

For the Street Name Corpus, our coherence score cross-validation yielded a 35-topic solution (see Figure 5; note that we have added the .HTML files for Figures 3 and 5 in [31]). The top 10 keywords for each topic are shown in Table 4, as is the number of documents sorted into each topic. We observed that content comprising each topic was notably more diverse and often did not pertain exclusively to drug use communication. For example, words like “skippy” and “vike,” which are common street names for Adderall and Vicodin, often have large amounts of crossover with tweets using these terms in other popular contexts including peanut butter (Skippy) and the Minnesota Vikings (Vikes). We can also directly observe this crossover when comparing the keywords in topics 34 and 35, where “barb” referred both to Nicki Minaj’s fanbase (eg, tweet: @NICKIMINAJ BARBS STAND TF UP) and actual barbiturate use (eg, tweet: Gotta pop this barb and take off tonight) with near equal frequency. We further observed that, beyond popular culture references, other street names queried for the analysis yielded references to securities and stock exchanges, as seen in topics 3 and 34, where oxy both refers to Oxycontin, the medication, as well as the publicly traded company Occidental Petroleum Corporation, whose stock listing is Oxy. In contexts in which the street medication did refer to a particular drug or substance, we observed colloquial-style communication and references to recreational use. Topics 21, 32, 13, and 14 show this phenomenon, while topics 3, 24, 33, and 34 show how those same terms can also be used to collect tweets about non-drug-related topics. Overall, we found that 34.3% (69,995/204,068) of all topics were related to a diverse use or multiuse of a word. We found that 26% (53,058/204,068) of topics referenced Percocet use, and 20% (40,814/204,068) of topics referenced different opioids.

**Table 4:** Street name topics (n=35).

Topic theme	Topic IDs	Cumulative amount, n (%)
No coherent topic	5, 11, 30, 17	4 (13)
Percocet	3, 9, 12, 13, 19, 21, 22, 26, 32	9 (26)
Diverse (all)	4, 6, 7, 10, 18, 23, 24, 25, 28, 33, 34, 35	12 (34)
Diverse: vikes	5, 11, 25	3 (9)
Diverse: oxy	7, 25	2 (6)
Diverse: soccer	33	1 (3)
Diverse: barbs	18, 23, 34, 35	4 (11)
Diverse: band	28	1 (3)
Diverse: skippy	6	1 (3)
Opioids	1, 2, 8, 14, 15, 29, 31	7 (20)
Uppers (amphetamine, stimulants)	16, 20, 27	3 (9)

### Aim 3: What Differences Could Be Observed Between Themes Identified From Brand Versus Street Names of the Same Prescription Drugs?

When comparing the topics generated from the Street Name Corpus and Brand Name Corpus, we found that the generated topics in both data sets included a lot of noise. However, the type and style of noise were not uniform. For example, in the brand name data set, we observed noise via the politization of drug use and US society and occasionally misnomers, including references to Sonata as either a medication or vehicle. Although people mentioned recreational drug use in the Brand Name Corpus, references to single and polydrug use were more apparent in the Street Name Corpus. However, noise in the Street Name Corpus, which broadly alluded to diverse use of a single term or multiple terms, made identifying drug-related tweets notably more difficult. We also found less discussion about personal illicit drug use in the Brand Name Corpus, since names like “fentanyl” or “oxycontin” were often mentioned in relation to how the drugs are used or to political issues. In comparison, the street name data set was where personal illicit drug use was discussed more often and was less easily categorizable as a result. Tweets in these topics either contained some mention of drug use or the street name was also associated with non-drug-related entities, resulting in the search query picking up tweets that were not related to drug use.

## Discussion

### Principal Findings

This study found that people discussed prescription drug use differently depending on whether a brand name or street name was used. Brand name categories often contained political talking points, while street name categories occasionally referenced drug misuse, though multiple social uses for a term muddled topic clarity. Content in the Brand Name Corpus reflected discussion about the drug itself and less often reflected personal use. However, content in the Street Name Corpus was notably more diverse and resisted simple LDA categorization.

This study demonstrates distinct differences between tweets containing brand names and those containing street names of prescription drugs. It is plausible that these differences represent “silos” of discourse regarding drug use on Twitter: one in which Twitter users are creating and responding to content regarding the reciprocal impact of drug use on US politics and society or challenges in legally obtaining drugs in a shortage (brand names) and one in which Twitter users informally convene to discuss their experiences in using and obtaining prescription drugs recreationally.

Our findings reinforce the difficult nature of digital surveillance for important and timely health topics. Particularly for street names, there is complexity in interpreting and even identifying the salient meaning in tweets from a large corpus of documents. For example, one might argue that information or words only attain meaning “in relation” to context [32]. In other words, a tweet containing the word “vikes” is not universally interpreted as a text about Vicodin (eg, depending on the context and on people’s own lexicon, the word might mean many different things to different people). In contrast, the term “fentanyl” (from the Brand Name Corpus) has a clear meaning that is largely independent both of context and of people’s lexicon but can take on different political overtones and meanings depending on the surrounding context, intention, and even the recipients who are tagged in the tweet. Thus, in examining how data for the corpora were parsed differently, we speculate that we observed more clarity in drug use typology with brand names because any meaning that emerged discursively or from context primarily pertained to other words within the tweets aside from the drug term itself (eg, supportive or oppositional to a particular policy). In contrast, with street names, the drug use term itself often was ambiguous and achieved meaning in Twitter conversations based on the shared understanding of other users. A tool such as LDA is powerful because it can process large volumes of information with minimal input, but this study shows how drug use researchers must use care in using such unsupervised approaches to conduct digital epidemiology, especially when intending to learn about use behavior.

Highlighting this complexity, visualization via distance mapping was ideal for the brand name tweets, but it did not represent the street name tweets well. This difference can be explained by the singular use of brand names (as a drug-related reference) relative to the multiple ways street names can be used and contextualized. For example, skippy, a well-known street name for Adderall, was often used in the context of the Skippy peanut butter brand. Likewise, the noise surrounding the street name tweets may create challenges for scientific data exploration but serves a practical purpose on Twitter of diverting attention or avoiding the attention of authorities. More research is needed to further contextualize how street names are used in a clandestine yet open manner online and on social media.

Although researchers have used various techniques to identify drug-related messages online, the structure, content, and function of drug use and overdose information engagement networks on Twitter have not been well explored. This work quantifies the content and context of communication about prescription drugs on Twitter and increases the understanding of key themes in dissemination. A limitation of our analytic approach to identifying major drug use themes on Twitter is that it does not address individual tweet content specifically. To gain a full understanding of the identified themes, we recommend large-scale human coding of random samples of collected tweet data to support these findings. To better understand the role of structure in drug use tweet dissemination, next steps will explore follower networks in drug use and overdose communication on Twitter, applying social network analysis to determine the characteristics and positions of important players, similar to work that has been done around political communication [33]. In an era of rapid information access and dissemination, the combination of quick and targeted interventions oriented to promote helpful drug use information or influence and reduce the impact of negative drug use information is key, especially for vulnerable populations such as youth. This work has the potential to be used for surveillance and detection of harmful information and for appropriate education and dissemination of information to persons engaged in drug use content on Twitter. Understanding the actors participating in these conversations may help us identify and engage influential players to reach people where they are (on Twitter) and disseminate relevant, timely, and effective health communication.

### Limitations

Our study was subject to limitations we hope to address in future work. First, although our analysis pipeline is supported in the literature, we relied on entirely unsupervised NLP analyses to analyze our data. Although the findings uncovered by the LDA, cross-validation, and initial data exploration tools are likely valid, more sophisticated and supervised machine learning analyses may have yielded further nuance. Future research should consider revisiting our data with such tools, including running topic models using S-BERT or GPT vectors. However, we caution that these analyses should only be undertaken in circumstances in which data are already highly cleaned and devoid of any noise, which our analysis sought to identify for future research. Second, we acknowledge that our informal review of topics did not constitute an in-depth qualitative evaluation of each topic. It is likely that performing more robust qualitative analyses would likewise yield more nuanced findings. Last, our study was likewise limited by abrupt changes to Twitter’s administrative team and particularly its purchase by current CEO Elon Musk; these changes limited our ability to collect more data given the slow truncation of Twitter’s API. The future of Twitter for academic research remains uncertain; therefore, we recommend a similar study be conducted on social networking websites other than Twitter, including Instagram and Threads, TikTok (barring future congressional bans), Mastadon, and/or Spill - examples of a growing number of Twitter alternatives with increasing popularity.

### Conclusion

Drug use is widely discussed on social media. However, using a brand name or street name notably altered the content of a given social media post. Our findings largely confirm that drug communication fell into either politically charged discussions of drug use or the context of using drugs for medical or recreational purposes. Given the overwhelming nature of social media and social media as data, the wide presence of drug use disclosures online may promote drug use behaviors among vulnerable populations, including people with drug use disorders and adolescents.

## Abbreviations

API: application programming interface
LDA: latent Dirichlet allocation
NLP: natural language processing
